# Non-inferiority of reverse hybrid regimen versus standard concomitant regimen for *H. pylori* eradication in a randomized-controlled trial

**DOI:** 10.22088/cjim.14.4.687

**Published:** 2023

**Authors:** Seyed Mohammad Valizadeh Toosi, Iradj Maleki, Vahid Hosseini, Hajar Shokri-Afra

**Affiliations:** 1 Gut and Liver Research Center, Non-communicable Diseases Institute, Mazandaran University of Medical Sciences, Sari, Iran

**Keywords:** Concomitant, Eradication regimens, * Helicobacter pylori* infection, Reverse hybrid, Randomized controlled-trial.

## Abstract

**Background:**

*Helicobacter pylori* (*H. pylori*) infection is strongly related to peptic ulcer disease, chronic gastritis, and gastric malignancies. Therefore, *H. pylori* eradication is necessary in these cases. This study was aimed to compare the efficacy of 14-day reverse hybrid therapy with standard 14-day concomitant regimen for *H. pylori* eradication in Iran.

**Methods:**

Of the 317 patients with dyspepsia and *H. pylori* infection enrolled in the study, 153 and 164 patients were randomly assigned to reverse hybrid and concomitant groups, respectively. The reverse hybrid regimen containing pantoprazole, amoxicillin, clarithromycin, and metronidazole was taken every 12 hours in the first 7 days, however, Clarithromycin and Metronidazole were discontinued within the next 7 days. Patients in the concomitant group also received the same drugs for 14-day. Eradication confirmation tests were used 8 weeks after the end of treatments.

**Results:**

A crowd of 281 patients continued the trial until the end. *H. pylori* eradication rates based on intention to treat analysis were 71.2% (109/153) and 83.5% (137/164) in reverse hybrid and concomitant groups, respectively (*P* = 0.007). By the per-protocol analysis, rates of eradication were 85.8% (109/127) and 89% (137/154), respectively (*P* = 0.428). Severe side effects were few in both groups. More side effects were observed in concomitant group (*p* < 0.001), however, the severity of side effects was not statistically different between the two regimens (*P* = 0.314). Reverse hybrid regimen was better tolerated (98% vs. 91.5%, *P* = 0.009).

**Conclusion:**

Both 14-day reverse hybrid and concomitant regimens have a fair response rate in Iran.

The infection rate of* Helicobacter pylori *(*H. pylori*) is nearly half of the world's population, so it has become a major global health issue (1, 2). Chronic *H. pylori* infection is the main cause of peptic ulcer disease (gastric or duodenal ulcer), chronic gastritis, and gastric adenocarcinoma (3-5). The increasing rate of antibiotic resistance of H. pylori to previously efﬁcacious regimens is alarming, so ongoing modiﬁcation of therapeutic approaches is necessary (3, 6).

 We have been using numerous therapeutic regimens as the first line for *H. pylori* eradication therapy, including the standard triple therapy regimen of 7-, 10-, and 14-day, 14-day Bismuth-based quadruple therapy, concomitant regimens of 10- and 14-day, sequential therapy of 10- and 14-day, and 10- and 14-day hybrid regimen (7-12). The major goal of all *H. pylori* eradication regimen is to achieve an eradication rate above 90% to 95% (13). 

Despite the various regimens for *H. pylori* eradication, the effectiveness of these regimens has reduced over time due to *H. pylori* resistance to antimicrobial agents (14, 15). Therefore, it is necessary to introduce new treatment regimens for patients in the hope to achieve an acceptable rate of eradication, ease of use, acceptable compliance, and lower drug side effects.

In areas where *H. pylori* is highly resistant to Clarithromycin (above 15%), especially if there is dual resistance to Clarithromycin and Metronidazole due to the poor results of triple therapy, it is recommended that 14-day concomitant quadruple therapy or 14-day Bismuth-based regimens be considered as first-line regimens for *H. pylori* eradication (3, 16). In addition, there is an ongoing evidence of hybrid regimens reporting an average per-protocol eradication rate of 93.3% (82.5% to 98.5%), despite the two antibiotics Metronidazole and Clarithromycin used in this regimen (9, 10). However, hybrid regimens efficiency varies in different geographical regions depending on *H. pylori* drug resistance (3, 9-12, 17). Moreover, the complexity of the drug intake in hybrid regimens is a weakness of this treatment, because an amoxicillin plus proton pump inhibitor (PPI) is given in the first halftime of the treatment period. In the second halftime, two antibiotics (Metronidazole and Clarithromycin) are added to Amoxicillin and PPI (11).

Recently, in a new regimen for *H. pylori* eradication entitled reverse hybrid regimen, patients have been treated with four drugs in the first 7 days (Pantoprazole, Amoxicillin, Metronidazole, and Clarithromycin), and in the next 5 or 7 days, Pantoprazole and Amoxicillin were only continued (PACM-7 & PA- 7 regimen) (18). Given the acceptable eradication rate and easier drug consumption by patients in this regimen, as well as owing to all previous studies of the reverse hybrid regimen are from areas with low drug resistance to *H. pylori* infection (18-21), we purposed to evaluate the efficiency of this regimen in our region where* H. pylori* showed high resistance to Metronidazole and Clarithromycin (14, 15). Here, we investigated the eradication rate of the 14-day reverse hybrid regimen (PACM-7-day & PA- 7-day) and compared it with the 14-day standard concomitant regimen (PACM- 14-day).

## Methods

Among the patients with symptoms of dyspepsia referred to the gastroenterology clinic, upper GI endoscopy was done for 597 patients. Deciding to perform upper GI endoscopy was according to the presence of warning signs and symptoms in a patient with dyspepsia. All dyspeptic patients above 45 years who had alarming symptoms of weight loss, iron deficiency anemia, hematemesis or melena, dyspepsia refractory to PPI therapy, and/or a first-degree relative history of gastric cancer were candidates for upper GI endoscopy.

Inclusion criteria were ages between 18 and 80 years who were *H. pylori*-positive and had any endoscopic findings of gastric ulcer (GU), gastric erosion (GE), duodenal ulcer (DU), duodenal erosion (DE), and histologic evidence of intestinal metaplasia. Pregnant or nursing women, those with a history of upper GI surgery, major cardiac, lung, liver, and renal diseases, history of previous *H. pylori* eradication regimen, and known side effects to the protocol drugs were excluded. According to the inclusion and exclusion criteria, finally, 317 patients were included and all of them signed the informed consent.

Endoscopy was performed by collaborating gastroenterologists using a Pentax endoscope (Version: EG2985). We evaluated *H. pylori* infection through two biopsies taken from the antrum and body by rapid urease test (RUT). Patients who had abnormal endoscopic findings were sampled from their visible abnormal lesions for histological examination. 

Patients were randomly allocated into two *H. pylori* eradication regimens. Based on the simple randomization method a shuffled deck of number cards was used. By default, the even and odd numbers were allocated to reverse hybrid and concomitant groups, respectively. In the 14-day reverse hybrid regimen, patients were offered to use four drugs (Pantoprazole 40 mg, Amoxicillin 1 g; Clarithromycin 500 mg, and Metronidazole 500 mg every 12 hours) in the first 7 days. Within the second 7 days, they were instructed to continue only pantoprazole and amoxicillin at the same dose and discontinue Clarithromycin and Metronidazole (PACM- 7-day & PA- 7-day). In the standard 14-day concomitant regimen, patients have taken the same dose of the above four drugs for 14-day (PACM- 14-day).

For each patient, a questionnaire including demographic information of age, sex, history of aspirin and NSAID usage, cigarette smoking, endoscopic findings as well as pathology results, and data of *H. pylori* infection was completed. During treatment, drug side effects including bitter taste, nausea, vomiting, diarrhea, pruritus, and other were recorded for all patients. Compliance of patients for drug consumption was assessed during the study. Patients with excellent compliance had consumed more than 80% of prescribed drugs. Those who took 60% to 80% of the drugs were considered intermediate compliance, and those who consumed less than 60% were categorized as low compliance. Eight weeks after treatment, the eradication was confirmed by *H. pylori* stool antigen test, and/or ^14^C- urease breathe test (UBT) and/or, pathology in patients who needed re-endoscopy.

Data were analyzed by Statistical Package for Social Sciences (SPSS) 22.0 software (IBM, Armonk, NY, USA). Descriptive statistics were applied to summarize the demographic data. Chi-square test and student’s t-test were used for qualitative and quantitative data, respectively. Results are reported as frequency or means ± standard deviations (SD). *P*-values < 0.05 were considered statistically significant. To calculate eradication rate based on intention to treat, all patients who were initially included in the study were analyzed, and to calculate eradication rate based on per-protocol, only patients who followed all the steps of the study protocol and took more than 80% of the drug, have entered the statistical analysis. 

**Ethical approval:** The study protocol was approved by the Ethics Committee of Mazandaran University of Medical Sciences (IR.MAZUMS.IMAMHOSPITAL.REC.1396.1475) and was also registered in the Iranian Registry of Clinical Trials with the IRCT number IRCT2017081215510N3 (Link: https://en.irct.ir/trial/14761). 

## Results

Of the 317 enrolled patients, 153 cases were randomly assigned to the reverse hybrid regimen and 164 to the concomitant regimen. Sex distribution in both groups did not have a significant difference. Females consisted of 62.3% and 51.8% of patients in reverse hybrid and concomitant groups, respectively (*P* = 0.08). The age distribution of patients in both groups was similar (44.9 ± 13.8 years and 46.3 ± 14.6 in both groups, *P* = 0.39). There was also no significant difference between the two groups in the usage of Aspirin, NSAIDs, and history of smoking (table 1). 

The most common endoscopic finding in all patients was peptic ulcer disease (GU and DU) (table 2). As shown in table 3, *H. pylori* infection was diagnosed in most patients (*n* = 189) by the RUT method confirmed with pathology. 

Regarding to the side effects of the drugs, 40.5% in the reverse hybrid regimen and 33.5% in the concomitant regimen did not mention any drug side effects (*P* < 0.001). Bitter taste was found as the most common side effect in the two groups (45% vs. 29.3%). Other drugs’ side effects including nausea, vomiting, diarrhea, and pruritus were less common in both regimens. The side effects of 50 patients from the concomitant group were not available. Patients mostly experienced mild forms of drug’s side effects and severe side effects were reported rarely. The compliance for drug consumption in both regimens was very good (98% and 91.5% for reverse hybrid and concomitant, respectively, *P* = 0.009) (table 4).

**Figure 1:**
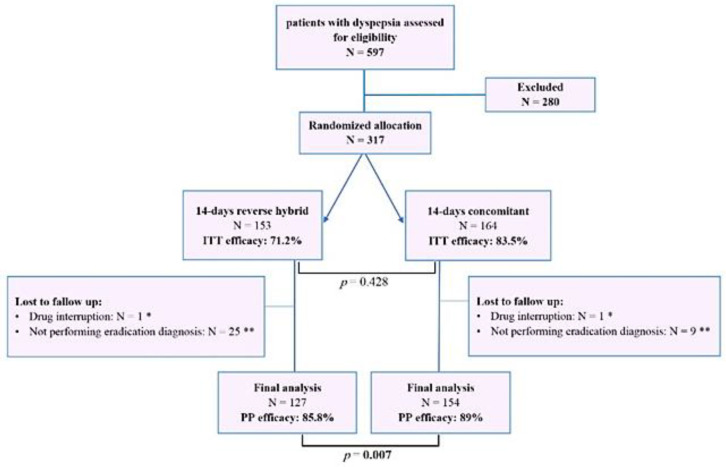
Method of follow-up and treatment efficac*y* for *H. pylori* eradication results of 14-day reverse hybrid and 14-day concomitant regimens. N: Number, ITT: intention to treat, PP: per-protocol. * These patients are among those who had severe side effects and did not have excellent compliance to treatment. ** These patients are among those who had excellent compliance to treatment without any side effects. The statistical difference between the two therapy groups is shown as a *p*-value. The significant *p*-value less than 0.05 is shown in bold

36 (26 from the reverse hybrid group and 10 from the concomitant group) patients did not complete the study. The reason for these patients' exclusion was their lack of cooperation in the final evaluation of *H. pylori* eradication at the end of treatment as well as having not excellent compliance due to severe adverse effects (figure 1). Confirmation of the* H. pylori* eradication was performed by UBT (*n* = 64), Fecal Ag (*n* = 188), and pathology (*n* = 29). The *H. pylori* eradication rate by intention to treat analysis was 71.2% (109/153) for reverse hybrid and 83.5% (137/164) for the concomitant regimen with a significant difference (*P* = 0.007). Based on the per-protocol analysis, *H. pylori* eradication rate for reverse hybrid and concomitant regimens were 85.8% (109/127) and 89% (137/154), respectively (*P* = 0.428) (figure 1). 

**Table 1:** Demographic data of 14-day reverse hybrid and 14-day concomitant regimens

**P-value**	**Concomitant group**	**Revers hybrid group**	
0.40	46.4 ± 14.7	45.0 ± 14.2	Age (mean ± SD)
**0.05**	79 (48.2)	57 (37.3)	Sex (male) (%)
	85 (51.8)	96 (62.7)	Sex (female) (%)
**0.05**	28 (17.1)	43 (27.9)	NSAID (%)
0.10	20 (12.2)	32 (20.8)	ASA (%)
0.08	18 (11)	27 (17.5)	Smoking (%)

The statistically significant difference with a *p*-value less than 0.05 are shown in bold.

**Table 2:** Endoscopic and pathologic data of 14-day reverse hybrid and 14-day concomitant regimens

Total	Concomitant group	Revers hybrid group	EGD findings
**90**	50	40	Gastric ulcer (GU)
**49**	29	20	Duodenal ulcer (DU)
**33**	16	17	DU + GU
**96**	44	52	Gastric erosion (GE)
**39**	19	20	Duodenal erosion (DE)
**10**	6	4	GE + DE

EGD: Esophagogastroduodenoscopy

**Table 3:** Methods of *H. pylori* diagnosis of 14-day reverse hybrid and 14-day concomitant regimens

**Total**	**Concomitant group**	**Revers hybrid group**	**Diagnosis**
31	12	19	RUT
5	3	2	RUT + serology
92	63	29	Pathology
189	86	103	RUT + pathology

RUT: rapid urease test

**Table 4:** Frequency and severity of side effects of 14-day reverse hybrid and 14-day concomitant regimens

P-value	Concomitant group	Revers hybrid group	
< 0.001			Side effects (%)
	55/164 (33.5)	62/153 (40.5)	- Without side effects
	48/164 (29.3)	69/153 (45.1)	- Bitter taste
	11/164 (6.7)	22/153 (14.4)	- Other side effects
	50/164 (30.5)	0/153 (0)	- Unknown
**0.314**			Severity of side effects (%)
	48/59 (81.4)	79/91 (86.8)	- Mild
	8/59 (13.6)	11/91 (12.1)	- Moderate
	3/59 (5.1)	1/91 (1.1)	- Severe
0.009	150/164 (91.5)	150/153 (98)	Drug compliance (%)

The statistically significant difference with a *p*-value less than 0.05 are shown in bold.

## Discussion

With increasing drug resistance in *H. pylori* eradication regimens, the success rate of these regimens has decreased over time. According to this fact, since* H. pylori* infection becomes difficult to treat, successful treatment requires the simultaneous administration of two or more antimicrobial agents. Drugs most commonly used to eradicate *H. pylori* infection include Metronidazole, Amoxicillin, Bismuth, Tetracycline, Clarithromycin along with PPIs (5). In concomitant and hybrid regimens, the type of antibiotics used is similar. Considering that these regimens had acceptable results in the eradication of *H. pylori *infection in previous studies (10-12), this study was aimed to investigate the efficacy of reverse hybrid regimen by simplifying the patients' drug intake.

This is the first study on the *H. pylori* eradication field in a region with dual antibiotic resistance to Metronidazole and Clarithromycin (14, 15) in which a 14-day reverse hybrid regimen was compared with a standard 14-day quadruple therapy. In this study, *H. pylori* eradication rate based on the per-protocol analysis was 85.8% and 89% in the reverse hybrid and concomitant regimens, respectively. According to an evidence-based study by Graham et al. the success rate of each regimen for *H. pylori* eradication (based on the per-protocol analysis) was classified as: eradication rate ≥ 95% as excellent success, 90-95% as good success, 85-89% as relatively good (acceptable borderline), and < 85% is considered unacceptable borderline (13). Therefore, as our results, the *H. pylori* eradication rate was relatively good in both regimens.

Hsu et al. in 2015 reported that Taiwanese patients who received standard triple therapy had a lower eradication rate than those treated by 12-day reverse hybrid (per-protocol analysis 88.3% vs. 95.7%; *P *= 0.005) (18). He and his colleagues also presented the effectiveness of the 14-day reverse hybrid regimen in 2018 with an *H. pylori* eradication rate of 96.6% (19). In line with previous studies, three other new studies reported similar results in the eradication of *H. pylori* by reverse hybrid regimen (> 95%) (20-22).We speculate that our lower success rate of *H. pylori* eradication by the reverse hybrid regimen compared to the abovementioned studies is probably due to the higher antibiotic resistance of *H. pylori* in our region. Because all the above studies are related to geographical areas with low drug resistance in *H. pylori* infection, therefore, their achievement of good to excellent eradication rates was to be expected. However, our relatively good success was also impressive despite the high antibiotic resistance of *H. pylori* in our region. In this regard, Lin et al. displayed that per-protocol *H. pylori* eradication rate in reverse hybrid regimen was 96.6% (a Taiwanese study). In sub-group analysis, for those patients who had single Clarithromycin resistance and dual resistance to Clarithromycin and Metronidazole, the rate of *H. pylori* eradication declined to 86.7% and 87.5%, respectively, which was close to our results. Therefore, they recommended that the 14-day reverse hybrid regimen could be applicable in areas with either high Clarithromycin or Metronidazole resistance (21). These explanations may justify the reason for the acceptable borderline *H. pylori* eradication during our study. However, the reverse hybrid therapy application for *H. pylori* treatment in regions with high resistance rates to Clarithromycin- and Metronidazole-containing regimens is not fully explored (23).

There are other regimens based on Clarithromycin and Metronidazole, such as concomitant, whose therapeutic effect has been confirmed in areas with either high Clarithromycin or Metronidazole resistance. Maastricht V Consensus Report has recommended concomitant therapy as the most effective quadruple therapy in the prevalence of dual antibiotic resistance to metronidazole and clarithromycin (3). We have previously showed the appropriate efficiency of these antibiotics in our region by concomitant regimen (12, 24, 25). Current results in line with our previous results, indicating that concomitant therapy could still be considered a relatively acceptable treatment in this region. Generally, to clarify the impact of antibiotic resistance on the eradication rate in areas with a high rate of antibiotic resistance, further investigations are suggested in the future.

The rates of total side effects were statistically lower in reverse hybrid therapy (55.5% vs. 66.5% *p* < 0.001), however, severe side effects were comparable between the two groups (*P* = 0.314). Bitter taste was the most common drug side effect in the two groups. Although drug compliance was acceptable in both groups, the reverse hybrid therapy was better tolerated (98% vs. 91.5 %, *P* = 0.009). The reason may be the difficulty of taking more drugs in the concomitant treatment.The limitation of the current study was the unavailability of techniques for assessing antibiotics susceptibility. Nonetheless, the strength of our study was the large sample size (*n* = 317), therefore, we can correlate the results with our regional resistance pattern (Mazandaran) (26). Moreover, the current work provided the first report of the reverse hybrid regimen efficacy on *H pylori* eradication in Iran. One of the advantages of the reverse hybrid regimen was the fewer number of medications used and hence, the lower cost of treatment. In the present study, 14-day reverse hybrid and 14-day concomitant regimens achieved a borderline acceptable response rate with few severe side effects. Therefore, these therapies could be considered the first-line treatment of *H. pylori* eradication in areas with high antibiotic resistance. Although the concomitant regimen had a more ideal eradication rate, the reverse hybrid regimen had statically lower side effects and more compliance. 

## References

[1] Sgouras DN, Trang TTH, Yamaoka Y (2015). Pathogenesis of Helicobacter pylori infection. Helicobacter.

[2] Ayana SM, Swai B, Maro V, Kibiki GS (2014). Upper gastrointestinal endoscopic findings and prevalence of Helicobacter pylori infection among adult patients with dyspepsia in northern Tanzania. Tanzan J Health Res.

[3] Malfertheiner P, Megraud F, O'morain C (2017). Management of Helicobacter pylori infection—the Maastricht V/Florence consensus report. Gut.

[4] Leja M, Axon A, Brenner H (2016). Epidemiology of Helicobacter pylori infection. Helicobacter.

[5] Mégraud F, Bessède E, Varon C (2015). Helicobacter pylori infection and gastric carcinoma. Clin Microbiol Infect.

[6] Thung I, Aramin H, Vavinskaya V (2016). Review article: the global emergence of Helicobacter pylori antibiotic resistance. Aliment Pharmacol Ther.

[7] Gisbert J, Calvet X (2011). Review article: non‐bismuth quadruple (concomitant) therapy for eradication of Helicobater pylori. Aliment Pharmacol Ther.

[8] Gisbert JP, Calvet X, O'Connor A (2010). Sequential therapy for Helicobacter pylori eradication: a critical review. J Clin Gastroenterol.

[9] Song ZQ, Liu J, Zhou LY (2016). Hybrid therapy regimen for Helicobacter pylori eradication. Chin Med J.

[10] Sardarian H, Fakheri H, Hosseini V (2013). Comparison of Hybrid and Sequential Therapies for H elicobacter pylori Eradication in I ran: A Prospective Randomized Trial. Helicobacter.

[11] Metanat HA, Valizadeh SM, Fakheri H (2015). Comparison Between 10‐and 14‐Day Hybrid Regimens for Helicobacter pylori Eradication: A Randomized Clinical Trial. Helicobacter.

[12] Alhooei S, Fakheri HT, Hosseini V (2016). A Comparison between hybrid and concomitant regimens for helicobacter pylori eradication: a randomized clinical trial. Middle East J Dig Dis.

[13] Graham DY, Lee YC, Wu MS (2014). Rational Helicobacter pylori therapy: evidence-based medicine rather than medicine-based evidence. Clin Gastroenterol Hepatol.

[14] Farzi N, Yadegar A, Sadeghi A (2019). High prevalence of antibiotic resistance in Iranian Helicobacter pylori Isolates: importance of functional and mutational analysis of resistance genes and virulence genotyping. J Clin Med.

[15] Savoldi A, Carrara E, Graham DY, Conti M, Tacconelli E (2018). Prevalence of antibiotic resistance in Helicobacter pylori: a systematic review and meta-analysis in World Health Organization regions. Gastroenterology.

[16] Fallone CA, Chiba N, van Zanten SV (2016). The Toronto consensus for the treatment of Helicobacter pylori infection in adults. Gastroenterology.

[17] Wu JY, Hsu PI, Wu DC, Graham DY, Wang WM (2014). Feasibility of shortening 14‐day hybrid therapy while maintaining an excellent Helicobacter pylori eradication rate. Helicobacter.

[18] Hsu PI, Kao SS, Wu DC (2015). A randomized controlled study comparing reverse hybrid therapy and standard triple therapy for Helicobacter pylori infection. Medicine (Baltimore).

[19] Hsu PI, Tsay FW, Graham DY (2018). Equivalent efficacies of reverse hybrid and bismuth quadruple therapies in eradication of Helicobacter pylori infection in a randomized controlled trial. Clin Gastroenterol Hepatol.

[20] Chang SN, Shih YH, Lee WC (2022). 14‐day reverse hybrid therapy vs 7‐day concomitant therapy in the first‐line treatment of Helicobacter pylori infection. Adv Dig Med.

[21] Lin TF, Wu DC, Tsay FW (2020). Reverse hybrid therapy achieves a similar eradication rate as standard hybrid therapy for Helicobacter pylori infection. J Chin Med Assoc.

[22] Hsu PI, Tsay FW, Kao JY (2020). Equivalent efficacies of reverse hybrid and concomitant therapies in first‐line treatment of Helicobacter pylori infection. J Gastroenterol Hepatol.

[23] Hu Y, Ouyang Y, Zhu Y, Lu NH (2021). Reverse hybrid therapy for Helicobacter pylori eradication: A systematic review and meta‐analysis. Helicobacter.

[24] Bari Z, Fakheri H, Taghvaei T, Yaghoobi M (2020). A Comparison between 10-day and 12-day Concomitant regimens for helicobacter pylori eradication: a randomized clinical trial. Middle East J Dig Dis.

[25] Yadollahi B, Valizadeh Toosi SM, Bari Z (2022). Efficacy of 14-day concomitant quadruple therapy and 14-day high-dose dual therapy on H pylori eradication. Gastroenterol Hepatology Bed Bench.

[26] Golrokhi MM, Fakheri H, Haghshenas MR, Ahanjan M (2016). The determination of antibiotic resistance of Helicobacter pylori isolated from patients living in north of Iran (Sari). Univ J Microbiol Res.

